# YB1 participated in regulating mitochondrial activity through RNA replacement

**DOI:** 10.3389/fonc.2023.1145379

**Published:** 2023-03-23

**Authors:** Weipeng Gong, Song Zhang

**Affiliations:** ^1^ Department of Gastrointestinal Surgery, Shandong Cancer Hospital and Institute, Shandong First Medical University and Shandong Academy of Medical Sciences, Jinan, Shandong, China; ^2^ Shandong Provincial Key Laboratory of Radiation Oncology, Shandong Cancer Hospital and Institute, Shandong First Medical University, Jinan, Shandong, China

**Keywords:** YB1, mitochondria, apoptosis, HMGA1, autophagy

## Abstract

As a relic of ancient bacterial endosymbionts, mitochondria play a central role in cell metabolism, apoptosis, autophagy, and other processes. However, the function of mitochondria-derived nucleic acids in cellular signal transduction has not been fully elucidated. Here, our work has found that Y-box binding protein 1 (YB1) maintained cellular autophagy at a moderate level to inhibit mitochondrial oxidative phosphorylation. In addition, mitochondrial RNA was leaked into cytosol under starvation, accompanied by YB1 mitochondrial relocation, resulting in YB1-bound RNA replacement. The mRNAs encoded by oxidative phosphorylation (OXPHOS)-associated genes and oncogene HMGA1 (high-mobility group AT-hook 1) were competitively replaced by mitochondria-derived tRNAs. The increase of free OXPHOS mRNAs released from the YB1 complex enhanced mitochondrial activity through facilitating translation, but the stability of HMGA1 mRNA was impaired without the protection of YB1, both contributing to breast cancer cell apoptosis and reactive oxygen species production. Our finding not only provided a new potential target for breast cancer therapy but also shed new light on understanding the global landscape of cellular interactions between RNA-binding proteins and different RNA species.

## Introduction

Mitochondria own a circular 16.6-kb DNA genome encoding two ribosomal RNAs, 22 transfer RNAs, and 13 open reading frames for electron transport chain complex subunits (1). During energy production, mitochondria participate in numerous metabolic reactions, such as tricarboxylic acid cycle and oxidative phosphorylation, and some intermediate metabolites are indispensable for cell proliferation and survival (2), but some by-products, such as reactive oxygen species, are harmful for most cells (3, 4). In recent years, more and more evidence indicated that there is a close relationship between cancer occurrence and mitochondria. Various studies have shown that mitochondria play a vital role in maintaining cancer cell stemness and drug resistance because of the ability of this organelle to modify cell metabolism, allowing survival and avoiding apoptosis clearance of cancer cells (5). Additionally, cancer cells can hijack the mitochondria from immune cells *via* building physical nanotubes to evade immune surveillance (6). Therefore, diving into studying the mechanisms underlying mitochondria-mediated signal flux is of great significance for understanding cellular homeostasis and cancer pathogenesis.

Y box binding protein 1 (YB1) is a member of the highly conserved cold-shock domain protein family with multifunctional properties and located in the cytoplasm and nucleus (7). Because cold-shock domains always endow proteins with the RNA/DNA binding ability (8), YB1 was implicated in various cellular processes, including transcription regulation, RNA splicing, translation, and RNA stability (9). In recent years, it was found that YB1 was upregulated in many kinds of cancers and usually indicated dismal clinical outcomes for cancer patients (10). In breast cancer cells, YB1 was aberrantly upregulated in ER-positive stem-like cancer cells. Through directly interacting with ERα, YB1 promoted ERα degradation, resulting in cancer stem cell differentiation (11). Upon DNA damaging agent exposure, YB1 was phosphorylated at the serine 102 residue by ribosomal S6 kinase and transported into the nucleus from cytosol to initiate the transcription of a set of drug-resistant and DNA repair genes (12, 13). During cancer metastasis, YB1 enhances HIF1a protein expression by directly binding to and activating translation of HIF1a message RNA (14). In addition, the transcriptions of some cell adhesion and extracellular matrix interaction proteins were also directly targeted by YB1 (15). Benefiting from the development of the RNA-seq technique, it has become accessible for us to read RNA modifications at a single-nucleotide resolution. At the same time, the novel function of YB1, as an RNA modification “reader,” was also uncovered in a lot of cancer cells (16). Through interacting with other partners, m^5^C and m^6^A modifications on tRNAs or mRNAs can be recognized by YB1 (17, 18). It is believed that new YB1-targeted drugs for cancer therapy will get more attention on this research background.

According to endosymbiosis theory, mitochondria originated from procaryotic organisms, indicating that mitochondrial nucleic acid elements and gene expression mechanisms are different from those that worked in the eukaryotic nucleus (19). It has been reported that mitochondrial DNA release *via* the permeability transition pore can trigger cellular inflammation through activating the cGAS/STING signaling pathway (20), but the downstream effects of mitochondrial RNA accumulation in cytosol are still elusive. In our study, we have found that upregulated YB1 in breast cancer cells increased cell tolerance to environmental stress through promoting autophagy and evading apoptosis-mediated cell clearance. Serum starvation induced mitochondrial RNA leakage and initiated the transport of YB1 into mitochondria. These mitochondria-derived RNAs competitively replaced YB1 original bound transcripts and reduced their half-life time, resulting in cancer cell inhibition and mitochondrial dysfunction.

## Materials and methods

### Cell culture

MDA-MB-231 breast cancer cells and HEK293T cells were purchased from Shanghai Cell Collection, Chinese Academy of Sciences. The cells were cultured in Dulbecco’s modified Eagle’s medium (Gibco, USA) supplemented with 10% fetal bovine serum (Gibco, USA) at 37°C in a humidified atmosphere with 5% CO_2_.

### Quantitative real-time PCR

Briefly, total RNAs were extracted from cells or tissues using an RNA isolation kit (Yeasen Biotech Corporation, Shanghai, China), and the complementary DNA was synthesized with a Reverse Transcription kit (Yeasen, Shanghai, China) according to the manufacturer’s instructions. Each gene RNA expression level was detected in SYBR Master Mixture with specific primers (listed in **Supplemental Table 1**). The relative expression levels of genes were calculated using the △△Ct method, and GAPDH (glyceraldehyde-3-phosphate dehydrogenase) was used as an internal normalization.

### Western blot

Cells were harvested in Western blot/IP lysis buffer (Beyotime, Beijing, China) and subjected to sonication. Then, the protein lysate was separated in 12% SDS-PAGE and electrotransferred to a polyvinylidene fluoride (PVDF) membrane (Millipore, USA). After incubation in blocking solution (5% skim milk) for 2 h, the membrane was incubated with a primary antibody at 4°C overnight. Subsequently, the membrane was incubated with secondary antibodies conjugated with horseradish peroxidase (Yeasen, China) for 1 h at room temperature and the signal of protein bond was detected using ECL chromogenic solution (Yeasen, China).

### Cell proliferation assay

Around 3,000 cells were seeded in each well of a 96-well plate and subjected to different treatments. At different times after seeding, each well was added with 10 μl MTS substrate (Promega, USA), followed by incubation at 37°C for 2 h. Then, the light absorbance of the plate at 480 nm was recorded by a microplate reader.

For cell colony formation assays, 1,000 cells were seeded into each well of a six-well plate and cells allowed to proliferate for 1 week. After fixation with 4% paraformaldehyde, the cells were stained with crystal violet solution for 20 min and the cell number was counted under a microscope.

### Lentiviral transfection

The plasmids of psPAX, pMD2G, and shRNA-containing constructs (Vigene Biosciences, USA) were co-transfected into HEK293T cells simultaneously. At 48 h after transfection, the cell culture supernatants containing virus particles were collected and filtered with a 0.22-μm membrane. Then, the virus liquid was added to cancer cell culture medium, allowing virus infection for 24 h. The puromycin-resistant cancer cells were isolated through treating cells with 5 μg/ml of puromycin (MCE, USA) for 1 week. The shRNA-mediated target gene knockdown efficiency was demonstrated by Western blot and quantitative real-time PCR.

### Cell cycle

Cells were fixed with 70% ethanol and stored at −20°C overnight. After being rinsed with PBS, the cells were treated with RNase A (20 µg/ml) for 40 min at 37°C, followed by incubation with propidium iodide (Sangon, Shanghai, China) at 37°C for 20 min. Then, the fluorescence intensity for each sample was analyzed by flow cytometry (BD, USA).

### Cell apoptosis detection

After centrifugation, cells were collected and stained with FITC Annexin V and propidium iodide using cell apoptosis detection kit (Beyotime, China) according to the manufacturer’s instructions. After rinsing, the signals of cells were analyzed by flow cytometry (BD Biosciences, USA) immediately.

### Transwell assays

Around 4 × 10^4^ cells cultured in serum-free medium were seeded into the upper chamber (8-μm pore size, Corning, USA), and the bottom well was added with 1 ml complete culture medium. At 48 h after incubation, the cells were fixed with 4% paraformaldehyde and stained with crystal violet solution. ImageJ, an image analysis software, was used to count the cell numbers for each experiment.

### Wound-healing assays

For this assay, around 2 × 10^5^ cells/well were seeded into a 12-well plate, allowing cells to proliferate up to around 90% confluence. The cell monolayer was scratched using a tip and washed with serum-free medium to remove detached cells. Then, the cells were cultured in complete medium and photographed at indicated times.

### Mitochondrial activity detection and ROS investigation

Mitochondrial activity was detected using MitoTracker dye (Beyotime, Beijing, China) according to the manufacturer’s instructions. Briefly, cells were seeded into a six-well plate 1 day before detection, and 1 µl MitoTracker dye was added into 5 ml DMEM cell culture medium to prepare cell staining solution. After mixing well, the mixture was added into each well and incubated with cells at 37°C for 90 min. Then, the cells were subjected to flow cytometry detection and fluorescence photographing. The procedure for ROS investigation was similar to the abovementioned steps, and a cellular ROS level detection kit was purchased from Beyotime Biotechnology.

### Mitochondrial isolation

Mitochondria were isolated from cancer cells using a cellular mitochondria isolation kit (Beyotime, China) according to the manufacturer’s instructions with some modifications. After trypsin treatment, around 1 × 10^7^ cells were collected and rinsed with PBS for twice. Then, the cells were resuspended with 4 ml prechilled solution I (containing 1mM PMSF) and stored on ice for 10 min. Then, the cells were homogenized in a prechilled Dounce homogenizer and centrifuged at 800g for 10 min at 4°C to remove cell debris. Mitochondria were sedimented at 13,000g for 10 min at 4°C, and the mitochondria-removed supernatant was collected. Subsequently, the rough mitochondrial fraction was washed with prechilled solution I and centrifugated at 13,000g for 10 min again. Purified mitochondria were incubated with digitonin on ice for 30 min at the indicated concentration. After resuspension in 1 ml solution I, mitoplasts were collected through centrifugation at 18,000g for 10min at 4°C.

### Fluorescence *in situ* hybridization and immunofluorescence

RNA fluorescence *in situ* hybridization (FISH) was performed with an RNA FISH kit (RiboBio, Guangzhou, China) according to the manufacturer’s instructions with minor modifications. After MitoTracker staining, cells were fixed with 4% formaldehyde for 10 min at room temperature. Then, the cells were permeabilized with 70% ethanol at 4°C overnight and dehydrated through 80, 90, and 100% ethanol. After pre-hybridization at room temperature for 30 min, the cells were incubated with hybridization buffer containing a 40-nM RNA probe (Generay, Shanghai, China) at 75°C for 5 min to allow RNA denaturation. Then, the cells were subjected to hybridization overnight at room temperature. Finally, the slides were washed with 4× SSC, 2× SSC, and 1× SSC buffer successively at room temperature.

After the finishing RNA FISH process, the slides were blocked with blocking solution (PBS + 0.5% bovine serum albumin) at room temperature for 1 h and incubated with the indicated primary antibodies for 2 h. After washing with PBS, the secondary antibodies with fluorescence modifications (Thermo Fisher, USA) were added and incubated for another 2 h. Finally, the nucleus was stained with DAPI (RiboBio, China) at room temperature for 10 min for microscopic observation.

### RNA-associated immunoprecipitation

After centrifugation, around 1 × 10^7^ cells were collected and resuspended in 1 ml RNase-free RIPA lysis buffer (Beyotime, China) containing 1 U/µl RNase inhibitor (Promega, USA). After cell lysis, whole-cell lysate was subjected to centrifugation at 12,000g for 10 min and the supernatant was divided into two aliquots. One aliquot was incubated with YB1 antibodies (Abmart, Shanghai, China) overnight at 4°C, and another aliquot was added with equivalent IgG isoforms as control. After incubation, the anti-YB1–RNA complex was precipitated with Protein G Agarose beads (Beyotime, China). After washing with RIPA lysis buffer for five times, the RNA bound to YB1 proteins was purified from the sedimented Agarose beads using the TRIzol RNA isolation kit (Yeasen Biotech Co., Shanghai, China), followed by quantitative real-time PCR.

### Protein stability

To measure protein stability, cells were treated with cycloheximide (CHX, final concentration 100 μg/ml, MCE, USA) to block the translation process. Cell samples were collected at indicated time points. In addition, to exclude the influence of protease-mediated protein degradation, MG132 (final concentration 50 μg/ml, MCE, USA) was added into the cell culture medium to inhibit protease activity for 8 h. The inhibitory efficiency was detected by Western blot.

### Animal models

Around 5 × 10^6^ cancer cells suspended in 800 μl PBS were mixed with Matrigel (Yeasen, China) at a volume ratio of 3:1. Then, 200 μl cell mixture was administered into each 4-week-old female BALB/c nude mouse by subcutaneous injection (n = 5). The width and length of tumor burdens were monitored weekly. When the tumor length was larger than 1 cm, all of the mice were sacrificed and the solid tumors were collected for the next investigation.

### Online data mining

The evaluation of YB1 expression in breast clinical specimens and associated patient survival rate was achieved through referring to the GEPIA database (21) (http://gepia.cancer-pku.cn/), KM plotter (22) (http://kmplot.com/analysis/), TNM plotter database (23) (https://www.tnmplot.com/), GENT2 (24) (http://gent2.appex.kr/gent2/), and TIMER (http://timer.cistrome.org/) (25). A protein–RNA interaction model was established by SWISS-MODEL (https://swissmodel.expasy.org/) (26).

### Statistical analysis

Software GraphPad Prism 8 was used for data processing and analysis. Samples with two groups were analyzed with Student’s t test, and samples of more than two groups were calculated with one-way ANOVA. When p < 0.05, the results were regarded as statistically significant.

## Results

### The upregulation of YB1 in breast cancerous tissues was associated with poor clinical outcomes

**Figure 1:**
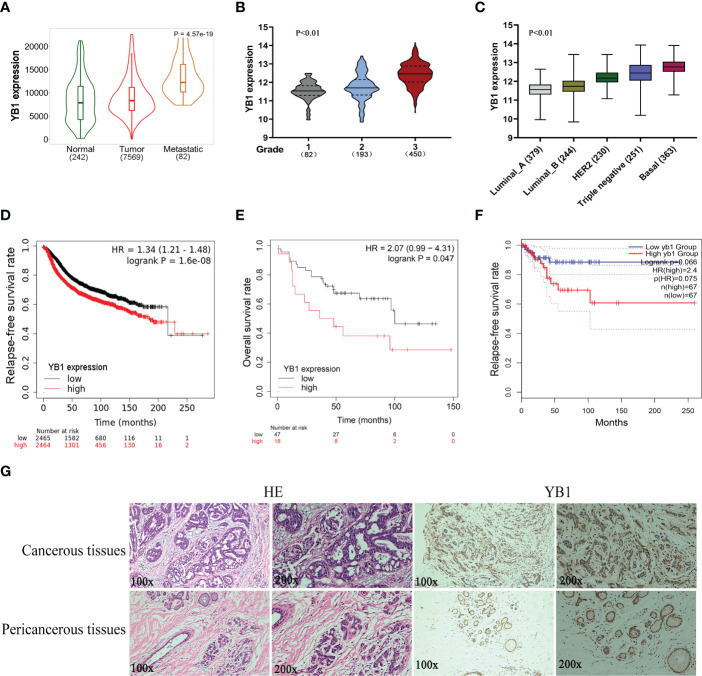
The upregulation of YB1 in breast cancerous tissues was associated with poor clinical outcomes. **(A)** Relative expression of YB1 in normal or cancerous tissues of patients with breast cancer. The expression level of YB1 in metastatic cancerous tissues was specifically elevated compared with normal or non-metastatic tissues (https://tnmplot.com). **(B)** Expression level of YB1 in breast cancer patients under different clinical stages (http://gent2.appex.kr/gent2/). **(C)** The relationship between YB1 expression and breast cancer types (http://gent2.appex.kr/gent2/). **(D-F)** Kaplan–Meier survival rates for patients with breast cancer stratified by YB1 expression. The survival rates for patients were plotted separately according to YB1 expression at the mRNA **(D)** or protein level **(E)** (http://kmplot.com/analysis/). The most remarkable difference emerged in patients with basal-like/triple-negative breast cancer **(F)**. **(G)** Immunohistochemical analysis of YB1 in solid tumors. Paired paracarcinoma and carcinoma tissues were excised from the same patient with breast cancer.

Previously, we have revealed the important role of YB1 in maintaining the stemness of melanoma stem cells, but the specific molecular mechanism whereby YB1 functions in breast cancer is still needed to be elucidated. Before exploring the function of YB1 in breast cancer, the relationship between YB1 and clinical outcomes was analyzed through referring to online databases. Compared with normal tissues, breast cancerous tissues showed higher YB1 expression, and the highest expression level of YB1 was presented in metastatic breast cancerous tissues (**Figure 1A**). In addition, the level of YB1 increased significantly with the aggravation of clinical staging (**Figure 1B**) and malignancy (**Figure 1C**). These data helped us build a close connection between YB1 expression and the progression of breast cancer. Benefiting from the availability of online databases, Kaplan–Meier survival rates for patients with low or high YB1 expression levels were plotted. No matter at the mRNA level or protein level, patients with lower YB1 expression usually have prolonged survival time compared with those with higher YB1 levels (**Figures 1D, E**), and this trend becomes more obvious especially for patients diagnosed with basal or triple-negative breast cancer (**Figure 1F**). Furthermore, immunohistochemical analysis showed that solid tumors have a higher YB1 expression level compared with pericancerous tissue (**Figure 1G**), which is consistent with the results from database. All the above mentioned data revealed YB1’s tumor-promoting nature and raised the potential of YB1 used as an indicator for breast cancer diagnosis.

### YB1 depletion inhibited breast cancer cell proliferation and migration

**Figure 2:**
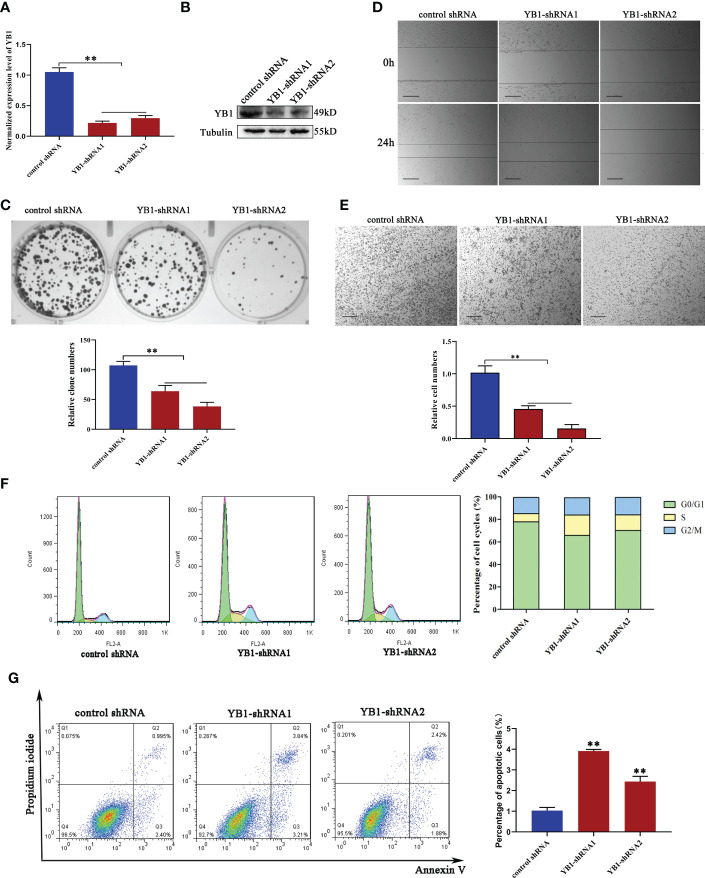
YB1 depletion inhibited breast cancer cell proliferation and migration. **(A, B)** Knockdown efficiency of YB1-targeted shRNAs in breast cancer cells (MDA-MB-231) at the mRNA level and protein level. β-Tubulin was used as a loading control. The experiment was repeated three times, **P < 0.01. **(C)** Colony formation assay was performed to investigate the influence of YB1 depletion on cancer cell proliferation. The experiment was repeated three times, **P < 0.01. **(D, E)** The function of YB1 on cell migration ability was investigated using wound-healing and Transwell assays. Scale bar, 100 μm. **(F)** YB1 expression silence induced obvious S-stage cell-cycle arrest in breast cancer cells. The cell cycle was investigated through flow cytometry. **(G)** The influence of YB1 knockdown on cell apoptosis detected by flow cytometry. Experiments were repeated three times.

In order to characterize the function of YB1 in breast cancer, breast cancer cell lines (MDA-MB-231) with stably decreased YB1 were established using lentivirus-mediated shRNA transfection. Quantitative real-time PCR and Western blot results demonstrated the good inhibitory efficiency of shRNAs on YB1 expression at the mRNA level and protein level (**Figures 2A, B**), respectively. Colony formation assays showed that the clonogenicity of breast cancer cells was significantly attenuated by YB1-silenced expression (**Figure 2C**). In addition, the role of YB1 in regulating cancer cell migration and invasion was also investigated using wound-healing and Transwell assays. The results showed that YB1 inhibition significantly impaired the invasion and migration ability of breast cancer cells (**Figures 2D, E**), further indicating the onco-promoting role of YB1. To further elucidate the mechanism of YB1-regulated cancer cell proliferation, cell-cycle and apoptosis levels were investigated. Flow cytometry analysis showed obvious S-stage cell-cycle arrest and increased apoptosis in cancer cells with decreased YB1 expression compared with the control groups (**Figures 2F, G**). All the above mentioned data indicated that YB1 functions as an oncogene in promoting breast cancer progression through regulating cell proliferation and migration.

### The role of YB1 in regulating cell apoptosis and autophagy

**Figure 3:**
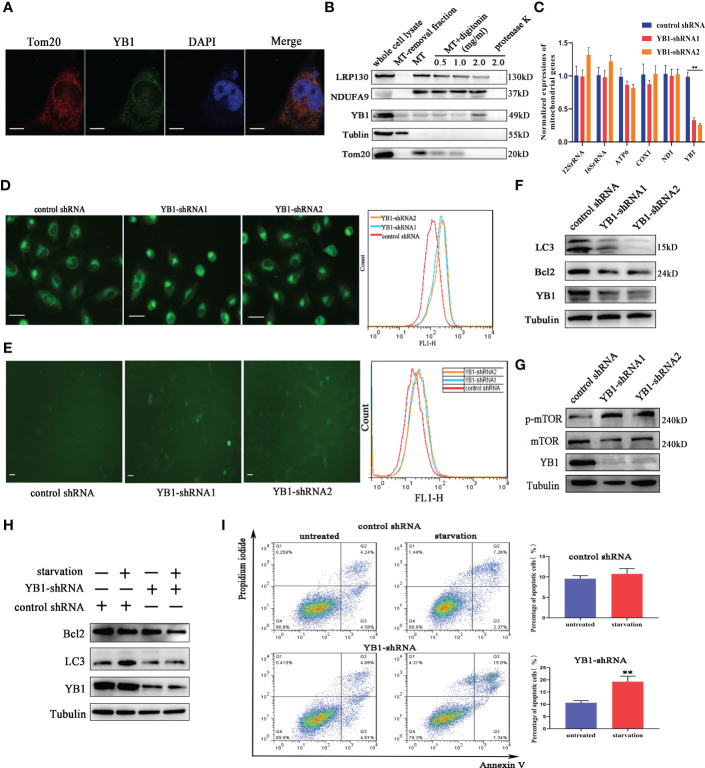
YB1 participated in balancing autophagy and cell apoptosis. **(A)** The co-localization of YB1 (green) with mitochondria (red) in breast cancer cells. Mitochondria were marked with TOM20 (red), and nuclei were stained with DAPI. Scale bars, 5 μm. **(B)** The protein level of YB1 in different mitochondrial fractions. Digitonin dose-dependently dissolved the mitochondrial outer membrane. The residual proteins were digested with protease K NDUFA9, inner membrane protein marker; LRPPRC, mitochondrial matrix localized protein. **(C)** Relative expressions of mitochondrial genome-encoded genes. The experiment was repeated three times. **p < 0.01. **(D)** The activity of mitochondria was investigated using flow cytometry. Mitochondria were labeled with MitoTracker dye; the fluorescence signal intensity was analyzed. Scale bar, 10 μm. **(E)** The influence of YB1 knockdown on cellular reactive oxygen species. Scale bar, 10 μm. **(F)** YB1 knockdown reduced the cellular basic autophagy level in breast cancer cells. **(G)** The mTOR signaling pathway was activated in cells with deleted YB1. **(H, I)** YB1-silenced expression failed to induce autophagy under starvation but significantly increased cell apoptosis.

To further explore the function of YB1 in breast cancer cells, the cellular distribution pattern of YB1 should be clarified. Here, we found that the majority of YB1 proteins was localized in cytoplasm. An obvious co-localization of YB1 with mitochondrial outer membrane marker TOM20 was also observed (**Figure 3A**). To figure out whether YB1 was imported into the mitochondrial matrix, the purified mitochondria were isolated from breast cancer cells using differential ultracentrifugation. Western blot results showed that YB1 existed in purified mitochondrial fraction even when the outer membrane was removed through digitonin treatment (**Figure 3B**), indicating that YB1 may localized to the mitochondrial matrix. The abovementioned data shed light on the potential interaction between YB1 and mitochondria; therefore, the influence of YB1 on mitochondrial activity was investigated. Quantitative real-time PCR data showed no difference in mitochondrial gene expressions in cancer cells with or without YB1 silence (**Figure 3C**). The status of mitochondria was investigated through loading the MitoTracker-specific probe into active cancer cells. It is surprising to find that YB1 depletion induced significantly enhanced mitochondrial membrane potential indicated by the evident fluorescence intensity shift (**Figure 3D**). In addition, the distribution of mitochondria was also disrupted by YB1 silence and more mitochondria were accumulated in the perinuclear region; fewer mitochondria localized to the cellular outer region compared with control cancer cells that showed randomly distributed mitochondria in cytoplasm (**Figure 3D**). In agreement with increased mitochondrial membrane potential, the levels of reactive oxygen species in YB1-silenced cells were also increased compared with the control group (**Figure 3E**). All of these data indicated the indispensable role of YB1 in regulating mitochondrial network formation in breast cancer cells.

Mitochondria are a cellular powerhouse that plays a fundamental role in energy production and cell survival, including cell apoptosis and autophagy. Here, Western blot results demonstrated that YB1 depletion significantly reduced the expression of LC3 (**Figure 3F**), a well-known protein marker for autophagy. By analyzing the innate signaling pathway participating in autophagy regulation, we found that the phosphorylation of mTOR was increased triggered by YB1 knockdown (**Figure 3G**), suggesting that YB1 might target mTOR for autophagy initiation. One of the well-established roles of mTOR is to promote anabolic cellular metabolism by suppressing autophagy. Considering that starvation can induce cell autophagy, we starved the cells through withdrawing serum from the cell culture medium for 24 h to determine if YB1 influences the homeostasis of mitochondria. Western blot results implied that starvation induced obvious autophagy in control cells, but not in cells with decreased YB1. In addition, YB1 knockdown impaired the expression of cell apoptosis inhibitors under starvation conditions (**Figure 3H**). Consistent with Western blot results, flow cytometry analysis demonstrated that starvation treatment induced cell apoptosis more easily in YB1-silenced cells than in control cells (**Figure 3I**), highlighting the important role of YB1 in balancing cell apoptosis and autophagy.

### YB1 directly interacted with mitochondrial RNA

**Figure 4:**
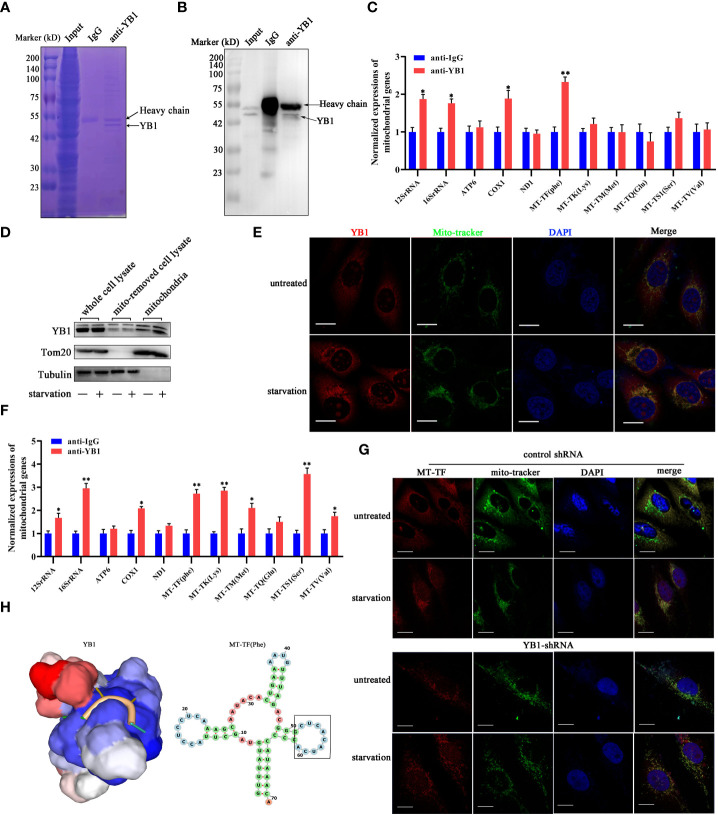
YB1 interacted with mitochondrial tRNA. **(A, B)** RNA-associated immunoprecipitation (RIP) assays. RIP was conducted in MDA-MB-231 cells to sediment the YB1–RNA complex; rabbit IgG was used as a negative control. The contents of immunoprecipitate were analyzed by SDS-PAGE with Coomassie staining **(A)** or Western blot **(B)**. **(C)** Detection of mitochondrial RNA in the YB1-immunoprecipitated complex. The RNAs co-immunoprecipitated with YB1 proteins were isolated and detected using quantitative real-time PCR. YB1 distribution was detected using the mitochondrial fraction **(D)** and immunofluorescence **(E)**; scale bar, 10 μm. **(F)** Serum-free starvation treatment increased the direct interaction between mitochondrial RNA and YB1 protein. n = 3, **p < 0.01, *p < 0.05. **(G)** MT-TF distribution detection using FISH. After starvation treatment, more MT-TF (red) were localized in the outside of mitochondria. Scale bar, 10 μm. **(H)** A YB1–RNA interaction model was built using the online SWISS-MODEL.

The function of YB-1 is underlain by its ability to interact with nucleic acids and proteins to form a heterocomplex. The co-localization of YB1 with mitochondria reminds us whether the RNA binding ability of YB1 was involved in regulating the activity of mitochondria. To elucidate this possibility, RNA-associated immunoprecipitation assays were performed to reveal the YB1-bound RNA library. Coomassie brilliant blue staining and Western blot results showed benign specificity of antibodies to sediment the YB1–RNA complex (**Figures 4A, B**). Quantitative real-time PCR revealed a strong interaction between YB1 and mitochondrial RNA, including 12S/16S rRNA, a fraction of mRNA, and transfer RNA (MT-TF) (**Figure 4C**). According to the data of quantitative real-time PCR, mitochondrial transfer RNA, MT-TF(phe) with the highest confidence, was picked up and focused in the next studies.

Subsequently, in order to investigate whether starvation treatment can influence the interaction between YB1 and mitochondrial RNA, mitochondria were isolated from cancer cells cultured under normal or starvation conditions. Western blot results showed an increased amount of YB1 in the purified mitochondrial fraction that underwent starvation (**Figure 4D**), consistent with the results of immunofluorescence staining (**Figure 4E**), indicating the transfer of YB1 into mitochondria. In addition, it is interesting to find that the amount of mitochondrial RNA was significantly increased in YB1 immunoprecipitates after starvation induction (**Figure 4F**). Additionally, microscope analysis observed leakage of mitochondrial RNA (MT-TF) into the cytoplasm in response to starvation treatment, which was further augmented in cancer cells with decreased YB1 expression (**Figure 4G**). Next, the protein–RNA interaction model was predicted using an online tool (https://swissmodel.expasy.org/). According to the secondary structure of MT-TF(Phe), a YB1 recognition motif (UCACAU) was positioned to a loop, which greatly increased the RNA accessibility for YB1 binding (**Figure 4H**). Collectively, these data indicated that starvation triggered the release of mitochondrial RNA into cytosol and mitochondrial import of YB1, which facilitated the interaction between YB1 protein and mitochondrial RNA.

### YB1 maintained the stability of HMGA1 mRNA

**Figure 5:**
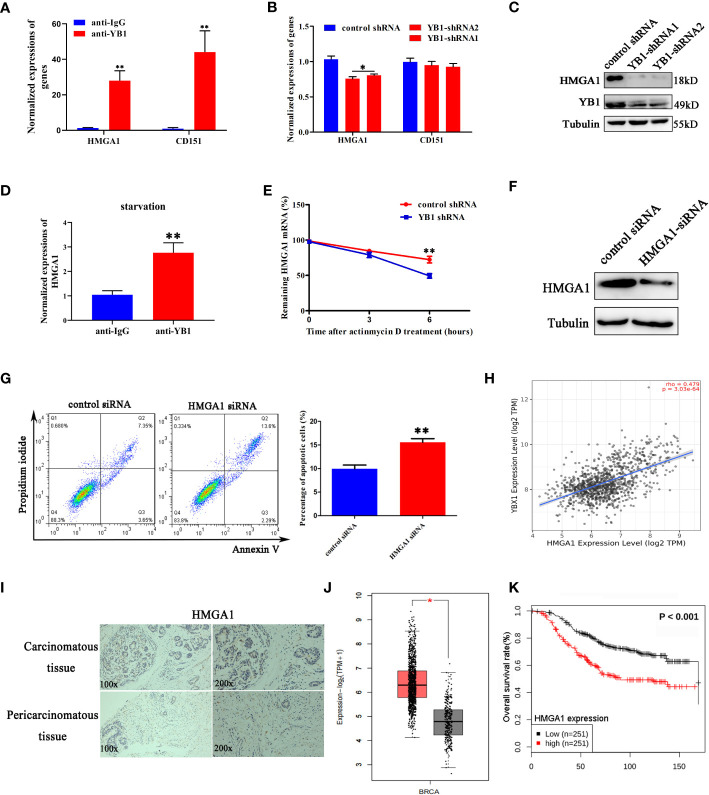
YB1 maintained the stability of HMGA1 mRNA. **(A)** Detection of oncogenes in the YB1-bound RNA library through RIP assays. **(B, C)** YB1 knockdown significantly downregulated HMGA1 expression at both mRNA level and protein level. **(D)** Serum-free starvation impaired YB1 binding ability with HMGA1 mRNA. n = 3,**p < 0.01. **(E)** Influence of YB1 knockdown on HMGA1 mRNA degradation. **(F)** The expression of HMGA1 was silenced using siRNA. **(G)** Influence of HMGA1 knockdown on cell apoptosis. n = 3, **p < 0.01. **(H)** Correlation analysis of YB1 expression with HMGA1 expression in breast cancer tissues. Data were downloaded from website http://timer.comp-genomics.org. **(I)** Immunohistochemical analysis of HMGA1 in paired breast carcinoma and paracarcinoma tissues. **(J)** Analysis of HMGA1 expression in breast cancer tissues and normal tissues through referring to an online database (http://gepia.cancer-pku.cn/). *p < 0.05. **(K)** High expression of HMGA1 in breast cancer tissues predicts adverse clinical survival outcomes (http://kmplot.com/analysis/).

Previously, a model has been proposed in which stress-induced transfer RNA fragments would be actively competing with endogenous transcripts for YB1 binding, resulting in RNA displacement and posttranscriptional regulation (27). In order to demonstrate the feasibility of this model in this cellular background, we need to clarify the species of YB1-bound endogenous transcripts. Through referring to others’ YB1-CLIP results (GSE63605), some onco-promoting gene mRNAs were found in the YB1-bound RNA library (27) and the expression levels of HMGA1 and CD151 were investigated in breast cancer cells. We have found that YB1 knockdown significantly reduced the expression of HMGA1 at the mRNA level and protein level (**Figures 5B, C**) but have no influence on CD151 expression, even though CD151 mRNA showed higher binding ability with YB1 protein (**Figure 5A**). In addition, serum-starvation treatment significantly impaired the interaction between YB1 and HMGA1 mRNA in breast cancer cells (**Figure 5D**). Compared with the control group, the half-life time of HMGA1 mRNA was significantly reduced in cancer cells with depleted YB1 expression (**Figure 5E**), indicating the important role of YB1 in regulating HMGA1 mRNA stability. To clarify whether HMGA1 was implicated in YB1-mediated cancer cell apoptosis, breast cancer cells were transfected with HMGA1-targeted siRNA and the knockdown efficiency was verified (**Figure 5F**). Flow cytometry analysis showed that HMGA1 silence induced significant cell apoptosis in breast cancer cells compared with the control group (**Figure 5G**). Correlational analysis through an interrogating online database (http://timer.comp-genomics.org/) showed a strong positive correlation between YB1 expression and HMGA1 expression in breast cancer tissues (**Figure 5H**). An elevated expression level of HMGA1 in cancer tissues and shorter patient survival time indicated the onco-promoting role of HMGA1 in breast cancer (**Figures 5I–K**). In summary, the cytosol accumulation of mitochondrial RNA and increased YB1 mitochondrial relocation induced by starvation changed YB1-bound original RNA species. Lacking YB1 protection enhanced HMGA1 mRNA degradation, which potentially induced cancer cell apoptosis and proliferation inhibition.

### YB1 inhibited OXPHOS gene translation

**Figure 6:**
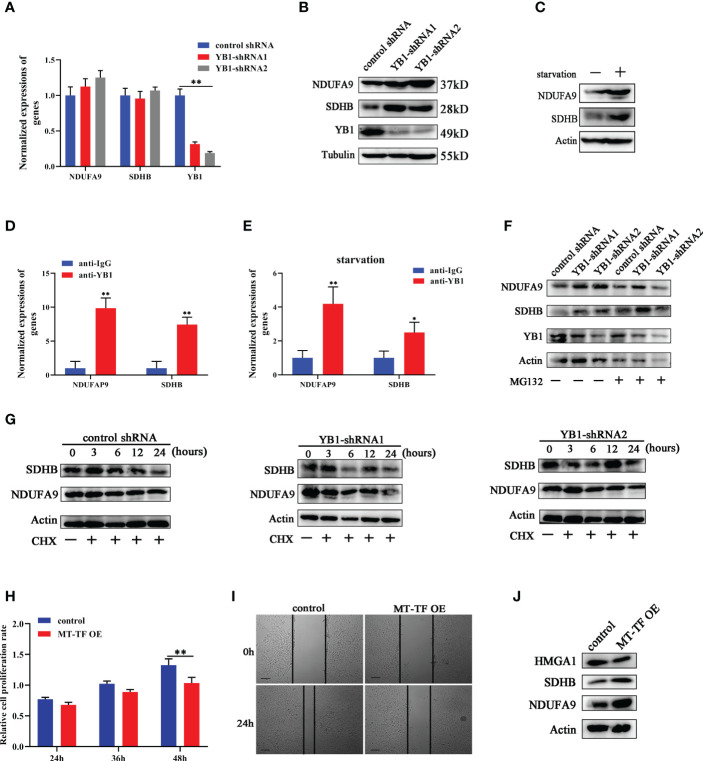
YB1 inhibited OXPHOS gene translation. **(A)** The influence of YB1 knockdown on mitochondrial oxidative phosphorylation gene (NDUFA9 and SDHB) expressions at the mRNA level. n = 3, **p < 0.01. **(B)** YB1 expression silence enhanced NDUFA9 and SDHB protein expressions through posttranscriptional regulation. **(C)** The influence of starvation on DNUFA9 and SDHB expressions was investigated through Western blot. **(D)** YB1 directly interacted with OXPHOS mRNA. The interaction between YB1 protein and OXPHOS mRNA (NDUFA9 and SDHB) was demonstrated using RIP assay. n = 3, **p < 0.01. **(E)** Serum-free starvation impaired YB1 binding ability with OXPHOS mRNAs. n = 3, *p < 0.05. **(F)** The protein levels of NDUFA9 and SDHB were investigated in breast cancer cells with or without YB1 depletion. MG132 was used to block the proteasome-mediated protein degradation pathway. **(G)** The protein degradation rates of NDUFA9 and SDHB were investigated. CHX was used to block the elongation phase of eukaryotic translation. **(H)** The influence of MT-TF overexpression (MT-TF OE) on breast cancer proliferation was detected using MTT assay. **(I)** The migration ability of breast cancer was inhibited after MT-TF overexpression. Scale bar, 100 μm. **(J)** The expressions of NDUFA9, SDHB, and HMGA1 were investigated in breast cancer cells transfected with MT-TF.

Except oncogene mRNAs, YB1 was reported to preferentially bind with oxidative phosphorylation (OXPHOS) mRNAs and suppressed their recruitment from inactive messenger ribonucleoprotein particles to active polysomes, leading to OXPHOS protein translation inhibition (28). Here, NDUFA9 and SDHB, as critical genes for oxidative phosphorylation, were selected for further analysis. Quantitative real-time RCR results showed that YB1 knockdown had no influence on NDUFA9 and SDHB mRNA expressions (**Figure 6A**) but significantly upregulated their protein levels (**Figure 6B**), which offered an explanation for our previous finding that YB1 knockdown increased mitochondrial membrane potential and the cellular ROS level (**Figures 3D, E**). Simultaneously, starvation treatment enhanced NDUFA9 and SDHB expression in cancer cells, but the total amount of YB1 showed no difference (**Figure 6C**). RIP assays demonstrated the direct interaction between YB1 and NDUFA9 and SDHB mRNAs, and this interaction was impaired after starvation treatment (**Figures 6D, E**), indicating the functional transformation of YB1. To exclude the influence of proteasome on YB1-mediated OXPHOS protein expression, breast cancer cells were treated with MG132, an inhibitor of 26S proteasome, to block protein degradation. Western blot results showed that MG132 treatment failed to block YB1-mediated NDUFA9 and SDHB upregulation (**Figure 6F**). In addition, protein stability assays demonstrated that YB1 silence had no influence on the degradation ratio of NDUFA9 and SDHB (**Figure 6G**). All of these data indicated that YB1 regulated OXPHOS-associated protein expression at the posttranscription level in a protease-independent manner, which is in accordance with others’ findings that YB1 functions as an inhibitor during OXPHOS gene translation (28).

To explore whether the RNA displacement model is suitable for OXPHOS expression, MT-TF was overexpressed in breast cancer cells through RNA transfection. Compared with the control group, MT-TF overexpression significantly inhibited breast cancer cell proliferation and migration (**Figures 6H, I**), which is consistent with the results induced by YB1 knockdown. Moreover, Western blot results demonstrated that MT-TF overexpression modestly increased the expression of NDUFA9 and SDHB but downregulated HMGA1 expression (**Figure 6J**), which indicated the universality of this model. This insignificant difference may be due to the inadequacy of transfected exogenous RNAs or the lack of an RNA secondary structure or modifications.

### YB1 promoted tumorigenesis of breast cancer cells

**Figure 7:**
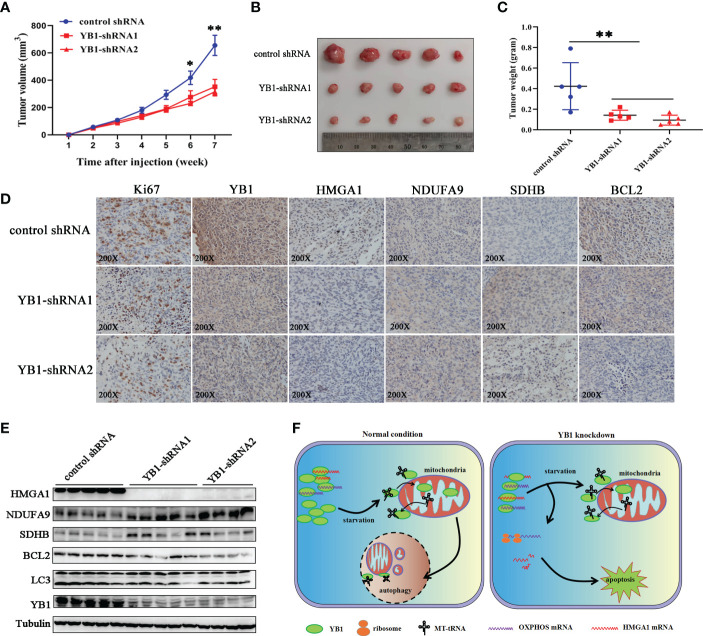
YB1 promoted tumorigenesis of breast cancer cells. **(A)** The statistics of xenograft volume in nude mice at different times. Breast cancer cells with different YB1 expression levels were subcutaneously injected into nude mice, and the volumes of tumors were monitored at different times. n = 5, *p < 0.05, **p < 0.01. **(B)** The representative pictures of solid tumors collected from nude mice. **(C)** Solid tumor weight analysis. n = 5, **p < 0.01. **(D)** Immunohistochemical analysis of YB-1-regulated proteins in breast cancer xenografts. Specific antibodies were used to detect each protein (brown), and nuclei were stained with hematoxylin (blue). **(E)** The levels of YB1-regulated proteins in xenografts were investigated through Western blot. **(F)** Proposed working model for the role of starvation-induced YB1-bound RNA replacement by mitochondrial RNA in regulating cell autophagy and apoptosis in breast cancer cells.

In order to explore the role of YB1 in cancer progression, breast cancer cells with stably silenced YB1 were subcutaneously injected into nude mice, followed by monitoring tumor growth every week for 7 weeks. Our results showed that YB1 knockdown significantly suppressed tumor growth *in vivo* compared with the control group (**Figure 7A**). After mouse sacrifice, the xenografts were collected and analyzed. Solid xenografted tumors with YB1 depletion showed smaller sizes and lower weights compared with the control group (**Figures 7B, C**), which revealed the positive role of YB1 in breast cancer progression.

In order to evaluate whether the RNA displacement model participated in regulating YB1-mediated cancer cell inhibition *in vivo*, the xenografts were subjected to immunohistochemical analysis. Compared with the control group, OXPHOS-associated factors (NDUFA9 and SDHB) showed an obvious expression increase in YB1-silenced tumors, but the expression of HMGA1 was decreased significantly (**Figure 7D**), suggesting the existence of an RNA displacement model in YB1-mediated breast cancer progression. In addition, according to Western blot investigation, LC3 and BCL2 were significantly downregulated in tumors with decreased YB1 (**Figure 7E**), but not in the control group, consistent with the finding *in vitro*.

Taken the abovementioned results together, we concluded that YB1 maintained cellular autophagy at a moderate level through binding with a lot of mitochondrial oxidative phosphorylation (OXPHOS) gene mRNAs to inhibit mitochondrial activity. Serum removal-mediated starvation increased the leakage of mitochondrial RNA into cytoplasm and YB1 mitochondrial relocation, resulting in YB1-bound RNA displacement. More OXPHOS and oncogene mRNAs were competitively replaced by MT-tRNA and then released from the YB1 complex. The increase of free OXPHOS mRNAs enhanced mitochondrial activity through strengthening protein translation, but the half-life time of free oncogene HMGA1 mRNA was impaired without the protection of YB1. All of these effects contributed to the downstream transformation from autophagy toward apoptosis in breast cancer cells (**Figure 7F**).

## Discussion

Our findings revealed that upregulated YB1 protein maintained cellular native autophagy at a high level through regulating the mTOR signaling pathway to enhance cancer cell tolerance for environmental stress. Loss-of-function experiments showed that YB1 deficiency alleviated cellular autophagy, enhanced mitochondrial oxidative phosphorylation complex protein translation, and thus augmented mitochondrial activity. The mechanism underlying YB1-mediated autophagy may be a result from the binding ability of YB1 with the p110β promoter to regulate its transcription (29). Additionally, it was found that loss of YB1 decreased the mRNA stability of Pink1 and Prkn in brown adipose tissue, two key regulators of mitophagy, leading to reduced protein expression, thereby alleviating autophagy (30). Owing to the uniqueness of different cells or tissues, more effects should be paid attention to elucidate the important role of YB1 in regulating autophagy.

Evidence has demonstrated that RNA-binding proteins could capture mRNA in the nucleus and form mRNPs to suppress transcript translation during nucleus–cytosol transport (31). Here, we found that a set of mitochondrial oxidative phosphorylation complex RNA transcripts, including NDUFA9 and SDHB, were present in the YB1–RNA heterocomplex and depletion of YB1 obviously increased oxidative phosphorylation complex protein translation, concomitant with others’ previous work. Through inhibiting mitochondrial functional protein production, YB1 may coordinate with mitochondrial homeostasis between autophagy and self-renewal. On whether YB1 directly interacts with these RNAs and whether other partners are indispensable during this process, some detailed issues have yet to be delineated, which defined a new direction for our next work.

An association between YB1 and transfer RNA fragments has been reported. Under serum starvation or hypoxic conditions, cells usually yield an amount of transfer RNA fragments in a short term and then suppress the stability of multiple oncogenic transcripts in breast cancer cells by displacing their 3′ untranslated regions from the RNA-binding protein YB1 (27). This kind of posttranscription regulation function mainly relied on the recognition of YB1 for the CU box motif enriched in targeted transcripts. Studies related to tRNA-mediated cellular translation suppression had also been reported in other cells and attributed to the disengagement of translational initiation factor EIF4G1 in a YB1-dependent manner (32). Here, our findings revealed that a class of distinct RNAs, mitochondria-derived RNAs, including MT-tRNA and ribosomal RNA, participated in the process of YB1-dependent transcript translation inhibition or RNA degradation regulation. Upon exposure to serum starvation, mitochondria-derived RNAs were accumulated in cytosol and this effect was reinforced especially in YB1-depleted cells. The transport of mitochondrial RNA into cytosol induced by starvation whether depending on specific mechanisms that are in charge of regulating permeabilization of mitochondrial membrane or just from fragmented mitochondrial debris is still unknown. During mitochondrial RNA release, some oncogenic transcripts were dissociated from the YB1 complex and their RNA decay rate was accelerated. However, according to our data regarding mitochondrial oxidative phosphorylation transcripts, YB1 inhibited these mRNA translations, which is contrary to the result of oncogenic transcripts, highlighting the complex and precise regulatory role of YB1 in balancing RNA translation and degradation. Given the extensive base modifications in tRNAs and the “reader” role of YB1 (17), further studies should be performed to decipher the interaction between YB1 and RNA at the single-nucleotide resolution level.

Taken together, we proposed an RNA displacement model in which YB1-bound RNAs were replaced by mitochondria-derived RNA under stress, resulting in targeted transcript release and induced different downstream effects. From a broader perspective, we speculated that a large regulatory network consisting of small ncRNAs and various RNA-binding proteins, not limited to YB1, orchestrated cellular homeostasis in a manner that is completely distinct from the traditional miRNA-mediated broad posttranscription regulation.

## Data availability statement

The original contributions presented in the study are included in the article/**Supplementary Material**. Further inquiries can be directed to the corresponding authors.

## Ethics statement

The studies involving human participants were reviewed and approved by The ethics committee of Shandong Cancer Hospital. The patients/participants provided their written informed consent to participate in this study. The animal study was reviewed and approved by The ethics committee of Shandong Cancer Hospital.

## Author contributions

All authors contributed to the article and approved the submitted version.
