# Supplementary material for: YB1 participated in regulating mitochondrial activity through RNA replacement
Source: Front Oncol. 2023 Mar 23;13:1145379. doi: 10.3389/fonc.2023.1145379 (PMC10076880; doi:10.3389/fonc.2023.1145379)
Supplement: Supplementary file 1 [file DataSheet_1.doc]

**Primers used for quantitative real-time PCR**

| YB1 | F:ATAGACCACGATTCCGCAGG  R:GGTAATTGAAGTTGCGGCGG |
| --- | --- |
| MT-TF | F:GTTTATGTAGCTTACCTCCT  R:TGTTTATGGGGTGATGTGAG |
| 12SrRNA | F:ACTGCTCGCCAGAACACTAC  R:GGTGAGGTTGATCGGGGTTT |
| 16SrRNA | F:CGATGGTGCAGCCGCTATTA  R:ATCATTTACGGGGGAAGGCG |
| GAPDH | F:GGTATCGTGGAAGGACTCATGAC  R:ATGCCAGTGAGCTTCCCGTTCAG |
| COX1 | F:ATACCAAACGCCCCTCTTCG  R:TGTTGAGGTTGCGGTCTGTT |
| ND1 | F:CGATTCCGCTACGACCAACT  R:AGGTTTGAGGGGGAATGCTG |
| CytoB | F: ACCCCCTAGGAATCACCTCC  R:GCCTAGGAGGTCTGGTGAGA |
| ATP6 | F:ACCACAAGGCACACCTACAC  R: TATTGCTAGGGTGGCGCTTC |
| CD151 | F: GTCATGGTGACTGGGGTCTT  R: TAGTAGGCGTAGGCGAGGAT |
| HMGA1 | F:GAGCTCGAAGTCCAGCCAG  R:CTCCTTCGGAGGCTGCTTG |
| SDHB | F:GTGGCCCCATGGTATTGGAT  R:CGGGTGCAAGCTAGAGTGTT |
| NDUFA9 | F:CCTTTGCTTTCGTTGGTCCC  R:CGATAGGCAAAAAGCGGCAA |

Probe used for RNA in situ hybridization assays:

MT-TF: Cy5-5’-GCCCGTCTAAACATTTTCAGTGTATTGCTT-3’

shRNA sequence used for RNA interference assays:

control shRNA: AGAGGACTTATCCGTTAGTC

YB1-shRNA1: AGCAGACCGTAACCATTATAG

YB1-shRNA2: CCAGTTCAAGGCAGTAAATAT

HMGA1-control siRNA:AUCCGAUACGUUAGAGCGA

HMGA1-siRNA: GACAAGGCUAACAUCCCAC

Antibodies information:

| Name | Company |
| --- | --- |
| YB1 | abcam (USA,ab76149) |
| β-Tublin | Cell Signaling Technology (USA) |
| β-Actin | Servicebio (China,GB15001) |
| NDUFA9 | ABclonal (USA, A3196) |
| SDHB | Beyotime (China, AF7956) |
| HMGA1 | Servicebio (China,GB113561) |
| LC3 | Beyotime (China, AL221 ) |
| BCL2 | Servicebio (China,GB113375) |
